# *Serratia marcescens*: an outbreak experience

**DOI:** 10.3389/fmicb.2014.00081

**Published:** 2014-03-06

**Authors:** Petra Gastmeier

**Affiliations:** Institute of Hygiene and Environmental Health, Charité - University Medicine BerlinBerlin, Germany

**Keywords:** *Serratia marcescens*, outbreak, neonates, infection control, multiresistance

One year ago, I had one of the worst experiences a hospital epidemiologist can have: a *Serratia marcescens* outbreak in a neonatal department with a total of 21 colonized or infected newborns. This outbreak caused headlines in all national TV channels and newspapers for at least one week in Germany.

What happened? On October 8th, we identified two newborns with *S. marcescens* bloodstream infection on the same day. The local health authorities were informed, the infected infants were isolated and staff was educated about the special risks of Serratia infections. In addition, environmental samples were taken to search for an external source and a general screening of all newborns of this neonatal intensive care unit was performed to identify possible additional colonized cases. After detecting further colonized patients, we extended the screening to two other neonatal wards and found more neonates colonized with *S. marcescens*. Because the units were unable to isolate all colonized neonates with the available staff, the hospital director decided to close the department for new admissions on October 18th. This was the reason why the media became interested and asked for an explanation. One colonized infant born with a severe heart defect was transferred to another hospital for heart surgery and died some days after the operation. The supposition of the media was that the infection had caused the infant's death and it was not as a result of the severe heart defect. As a consequence, the State Attorney's Office opened an investigation into negligent manslaughter by persons unknown.

Our experience is in strong contrast with the knowledge about *S. marcescens* about 50 years ago. Until the 1950s, microbiologists considered this pathogen a harmless saprophyte. Because of its red pigment it served as a tracer organism to identify the spread of other microorganisms such as influenza viruses. It was used in World War I and until 1968 for military experiments to investigate transmission of pathogens (Mahlen, 2011). The first description of lethal *S. marcescens* cases in newborns was published in 1961 (Urmenyi and Franklin, 1961). A report from our own institution from 1989 described a prolonged outbreak with 222 cases of neonatal septicemia and/or meningitis in the period between 1983 and 1988. The incidence was 8.46 per 1000 liveborn infants. The case fatality rate amounted to 45.9%. (Grauel et al., 1989).

Current data from the German national nosocomial surveillance system for very low birth weight (VLBW) infants with 234 neonatal units participating show that 1.2% of blood stream infections with an identified pathogen are due to *S. marcescens*. The incidence of nosocomial infections with *S. marcescens* was 1.1 per 1000 VLBW in the period from 2008 to 2012 (Nationales Referenzzentrum für die Surveillance von nosokomialen Infektionen Available online at: http://www.nrz-hygiene.de).

However, the proportion of *S. marcescens* infections is much higher when analyzing outbreak data. *S. marcescens* had the third highest number of published outbreaks following Klebsiella spp. and *S. aureus* (Gastmeier et al., 2007). In most of the published neonatal *S. marcescens* outbreaks, it was impossible to identify the source of the outbreak. A recent query of the Worldwide Database with more than 3000 nosocomial outbreaks published in the literature (www.outbreak-database.com) identified 109 *S. marcescens* outbreaks. Forty-eight of these outbreaks (44%) were described in neonatal units. The average number of cases in the neonatal outbreaks was 33 with a range from 4 to 159. In about 60% of *S. marcescens* outbreaks in neonatal departments, it was impossible to identify the source (Table 1).

**Table 1:** **Distribution of outbreak sources for neonatal *S. marcescens* outbreaks**.

**Source**	**Neonatal out breaks with *S. marcescens* (%)**
Index patient	8 (16.6)
Care equipment	5 (10.4)
Environment	4 (8.3)
Food	1 (2.1)
Medical equipment	1 (2.1)
Drug	0
Personnel	0
Unknown	29 (60.4)
Total	48 (100.0)

Of course, the published outbreaks are only the tip of the iceberg, and one can expect that at least 2–3 *S. marcescens* outbreaks occur annually in German neonatal intensive care units (Schwab et al., 2014).

One year later, the State Attorney's Office closed its investigation in Berlin and concluded on the basis of an autopsy by two pathologists that the death was not due to negligence (but rather because of the birth defect) and that were no cases of physical injury due to negligence. The hospital's infection control measures, they concluded, were appropriate. Among more than 600 environmental samples, we did not find any evidence for an environmental source. Looking back, it became clear that a mother with an amnion infection syndrome and identification of *S. marcescens* three months earlier was perhaps the source of the outbreak. She infected her infant and a further infant was colonized, but we did not find any other infected of colonized patients in the surrounding of these newborns. This child was also transferred to the heart surgery center and came back some weeks later.

One year after the outbreak, we can say that after identifying all colonized neonates in the first week of the outbreak by the extensive screening, no further neonates became infected although the last infant of the outbreak group was discharged 7 months later. In addition, scientists have shown by whole genome sequencing that that our *S. marcescens* strain had special virulence factors which lead to a rapid spread of this microorganism (submitted).

A recently published article analyzing fecal microbiota during the first month of life concluded that the presence of Serratia was strongly associated with a higher degree of immaturity and other hospital-related parameters, including antibiotic therapy and mechanical ventilation (Moles et al., 2013). This means that *S. marcescens* remains a dangerous pathogen in neonatal intensive care units. Our *S. marcescens* strain was a susceptible one, but the problem may even increase when resistant strains cause outbreaks. In 2013, the first outbreak with a Carbapenemase-producing *S. marcescens* was published in Argentina (Nastro et al., 2013).
